# Inflammation-associated extracellular β-glucuronidase alters cellular responses to the chemical carcinogen benzo[a]pyrene

**DOI:** 10.1007/s00204-015-1593-7

**Published:** 2015-10-05

**Authors:** Q. Shi, G. R. Haenen, L. Maas, V. M. Arlt, D. Spina, Y. Riffo Vasquez, E. Moonen, C. Veith, F. J. Van Schooten, R. W. L. Godschalk

**Affiliations:** 1 Department of Pharmacology and Toxicology, NUTRIM School of Nutrition and Translational Research in Metabolism, Maastricht University, PO Box 616, 6200 MD Maastricht, The Netherlands; 2 Analytical and Environmental Sciences Division, MRC-PHE Centre for Environmental and Health, King’s College London, 150 Stamford Street, London, SE1 9NH UK; 3 NIHR Health Protection Research Unit in Health Impact of Environmental Hazards at King’s College London in Partnership with Public Health England, 150 Stamford Street, London, SE1 9NH UK; 4 Sackler Institute of Pulmonary Pharmacology, Institute of Pharmaceutical Science, King’s College London, 150 Stamford Street, London, SE1 9NH UK

**Keywords:** Benzo[a]pyrene, Inflammation, β-Glucuronidase, Cytochrome P450 1A1, Carcinogen metabolism, IGF2R, DNA adducts

## Abstract

Neutrophils infiltrate tissues during inflammation, and when activated, they release β-glucuronidase. Since inflammation is associated with carcinogenesis, we investigated how extracellular β-glucuronidase changed the in vitro cellular response to the chemical carcinogen benzo(a)pyrene (B[a]P). For this we exposed human liver (HepG2) and lung (A549) cells to B[a]P in the presence or absence of β-glucuronidase. β-Glucuronidase reduced B[a]P-induced expression of *CYP1A1* and *CYP1B1* at 6 h after exposure, which did not depend on β-glucuronidase activity, because the inhibitor d-saccharic acid 1,4-lactone monohydrate did not antagonize the effect of β-glucuronidase. On the other hand, the inhibitory effect of β-glucuronidase on CYP expression was dependent on signalling via the insulin-like growth factor receptor (IGF2R, a known receptor for β-glucuronidase), because co-incubation with the IGF2R inhibitor mannose-6-phosphate completely abolished the effect of β-glucuronidase. Extracellular β-glucuronidase also reduced the formation of several B[a]P metabolites and B[a]P–DNA adducts. Interestingly, at 24 h of exposure, β-glucuronidase significantly enhanced *CYP* expression, probably because β-glucuronidase de-glucuronidated B[a]P metabolites, which continued to trigger the aryl hydrocarbon receptor (Ah receptor) and induced expression of *CYP1A1* (in both cell lines) and *CYP1B1* (in A549 only). Consequently, significantly higher concentrations of B[a]P metabolites and DNA adducts were found in β-glucuronidase-treated cells at 24 h. DNA adduct levels peaked at 48 h in cells that were exposed to B[a]P and treated with β-glucuronidase. Overall, these data show that β-glucuronidase alters the cellular response to B[a]P and ultimately enhances B[a]P-induced DNA adduct levels.

## Introduction

Chronic inflammation is causally associated with cancer development, and therefore, inflammation was considered as the seventh hallmark feature of cancer (Colotta et al. 2009; Shacter and Weitzman 2002). This is illustrated for instance by the relatively high incidence of lung cancer in chronic obstructive pulmonary disease (COPD) patients (Young et al. 2009). Recent studies have pointed out that polymorphonuclear neutrophils (PMN), which are recruited at the site of inflammation play an important role in the initiation and progression of cancer (Fridlender and Albelda 2012; Knaapen et al. 2006). Inhalation of complex air pollutants like tobacco smoke and/or fine particles may result in pulmonary inflammation, which generates reactive oxygen/nitrogen species (ROS/RNS) that can damage lung tissue (van Berlo et al. 2010). At the same time, it has been shown that under inflammatory conditions, PMN enhance the mutagenic potential of chemical carcinogens (Borm et al. 1997; Van Schooten et al. 2004). This is partly explained by the action of the PMN-derived enzyme myeloperoxidase (MPO) that can metabolically activate carcinogens and inhibit DNA repair, leading to higher levels of carcinogen–DNA adducts (Gungor et al. 2010a, b, c). However, there is still little data available on other factors that are released by the relatively high number of PMN during chronic inflammation, including β-glucuronidase, and how these influence the cellular response to chemical carcinogens (Basinska and Florianczyk 2003).

**Fig. 1:**
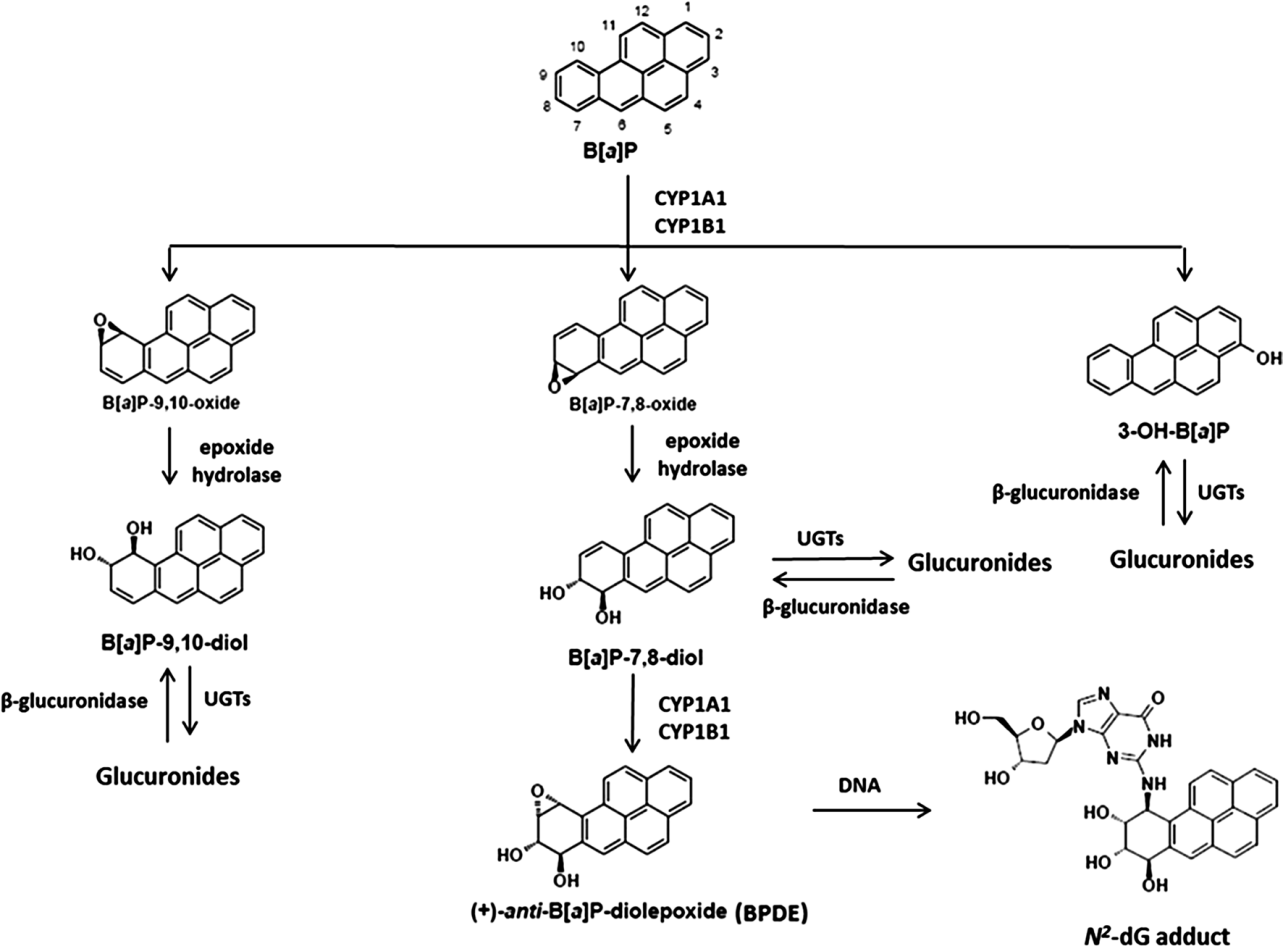
Critical steps of B[a]P activation and UDP-glucuronosyltransferases (UGTs) detoxification. B[a]P is metabolized to hydroxylated B[a]P including B[a]P-7,8-diol and these B[a]P metabolites are further detoxified by UGTs. β-Glucuronidases are able to hydrolyse glucuronidated B[a]P metabolites and therefore increase the amount of B[a]P-7,8-diol, consequently leading to more BPDE and DNA adducts formation

Inhalatory exposure to air pollutants like particles often results in a combination of an inflammatory response in the presence of genotoxic agents, like polycyclic aromatic hydrocarbons (PAHs) (Hoffmann and Hoffmann 1997). PAHs, including benzo[a]pyrene (B[a]P), have gained much attention, because they are abundantly present in the environment (Uppstad et al. 2010), and they have serious adverse genotoxic effects. B[a]P becomes mutagenic and carcinogenic after bioactivation by enzymes, including cytochrome P450 (CYP) and epoxide hydrolase (EH) (Stiborova et al. 2014). It is converted into various metabolites, including oxides, phenols, diols, diol-epoxides, quinones, and radical cations (Shimada and Guengerich 2006). The best studied metabolite is B[a]P-diol epoxide (BPDE) (Fig. 1). The first steps is the conversion of B[a]P by the microsomal NADPH-dependent cytochrome P450 isoforms 1A1 (CYP1A1) and 1B1 (CYP1B1) to yield the B[a]P-7,8-oxide, B[a]P-9,10-oxide or 3-hydroxy-B[a]P (3-OH-B[a]P) (Krais et al. 2015; Wohak et al. 2014). Subsequently, both B[a]P-7,8-oxide and B[a]P-9,10-oxide can be hydrated by microsomal EH to yield the corresponding B[a]P-7,8-*trans*-dihydrodiols (B[a]P-7,8-diol) and B[a]P-9,10-*trans*-dihydrodiols (B[a]P-9,10-diol). B[a]P-7,8-diol is further metabolized to the ultimate carcinogen benzo[a]pyrene-7,8-diol-9,10-epoxide (BPDE), which is known as a reactive derivative of B[a]P that can covalently bind to DNA to form adducts preferentially at guanine residues (e.g. 10-(deoxyguanosin-*N* ^2^-yI)-7,8,9-trihydroxy-7,8,9,10-tetrahydro benzo[a]pyrene; dG-*N* ^2^-BPDE) (Arlt et al. 2008; Kim et al. 1998; Wohak et al. 2014). It is generally accepted that the formation of such DNA adducts leads to mutations relevant for carcinogenesis (Kucab et al. 2015). In addition, the reactive metabolites can be conjugated by an important phase II detoxification enzyme UDP-glucuronosyltransferases (UGTs) that leads to glucuronides which are not mutagenic or carcinogenic (Shimoi and Nakayama 2005; Shimoi et al. 2001). Indeed, UGT1A6 was found to glucuronate a range of B[a]P metabolites including 3-OH-B[a]P, B[a]P-9,10-diol and the pro-carcinogen B[a]P-7,8-diol (Jin et al. 1993; Trushin et al. 2012; Zheng et al. 2002).

In order to elucidate the role of β-glucuronidase in inflammatory disease and its effect on chemically induced cancer, it is crucial to understand how β-glucuronidase changes the cellular response towards B[a]P. In a recent study, it was found that lipopolysaccharide (LPS) treatment increased B[a]P-induced DNA adduct levels in lung and liver tissues of B[a]P inhalatory-exposed mice (Arlt et al. 2015). To explain this result, we hypothesise that the LPS induced an inflammatory response resulting in the release of β-glucuronidase that hydrolysed glucuronidated B[a]P metabolites and thus reversing the protective effect of glucuronidation, and subsequently enhanced the binding of B[a]P metabolites to DNA. To test our hypothesis, we studied the impact of extracellular β-glucuronidase on the formation of B[a]P metabolites (3-OH-B[a]P, B[a]P-9,10-diol and B[a]P-7,8-diol), gene expression of some enzymes pivotal in B[a]P metabolism (including *CYP1A1*, *CYP1B1* and *UGT1A6*), and DNA adduct formation in human liver cell line (HepG2) and human lung cell line (A549).

## Materials and methods

### Mouse lung and liver tissues

All procedures performed in the study involving animal experiments were conducted at King’s College London under license in accordance with the Institutional Ethics Committee on the protocols approved by the Home Office under “The Animals (Scientific Procedures) Act (1986).” Mice were divided into four groups (*n* = 3 in each group): (a) control group; mice were nasally instilled with saline. After 24 h, mice were intratracheally instilled with tricaprylin. (b) The lipopolysaccharide (LPS) treatment group; each mouse was nasally instilled with 20 μg LPS (dissolved in saline). After 24 h, mice were intratracheally instilled with tricaprylin. (c) The B[a]P-treated group; mice were nasally instilled with saline and intratracheally instilled with 0.5 mg B[a]P (dissolved in 25 μl tricaprylin) after 24 h. (d) The B[a]P- and LPS-treated group; each mouse was nasally instilled with 20 μg of LPS. After 24 h, mice were intratracheally instilled with 0.5 mg B[a]P. All mice were sacrificed at 48 h after the intratracheal exposure. The collection of bronchoalveolar lavage fluid (BAL fluid) and the isolation of cytosolic fractions from lung and liver were performed as described in Arlt et al. (2015).

### Cell lines and cell treatment

Human liver hepatocellular carcinoma HepG2 cells and human epithelial lung adenoma carcinoma A549 cells were obtained from the American Tissue Culture Collection. HepG2 cells were cultured in minimum essential medium (MEM) plus glutamax containing 10 % (v/v) foetal calf serum (FCS, Gibco invitrogen, Breda, the Netherlands), 1 % (v/v) sodium pyruvate, 1 % (v/v) penicillin/streptomycin (Sigma, Zwijndrecht, the Netherlands), and 1 % (v/v) non-essential amino acids (Sigma). A549 cells were cultured in RPMI (Sigma) supplemented with 5 % (v/v) FCS and 1 % (v/v) penicillin/streptomycin. All cells were cultured under humidified atmosphere containing 5 % CO_2_ at 37 °C. Cell passages between 20 and 29 were used for experiments. All chemicals were purchased from Sigma-Aldrich unless stated otherwise.

Cells with 80 % confluency were exposed to 1 μM B[a]P in the presence or absence of β-glucuronidase (4 U/ml) for 6, 24 or 48 h. B[a]P was dissolved in dimethylsulphoxide (DMSO) and added to the medium with a final DMSO concentration of 0.5 % (v/v). β-Glucuronidase was dissolved in 0.1 M sodium acetate buffer (pH 5.5). Mannose-6-phosphate (M6P) was dissolved in 0.1 M sodium acetate buffer (pH 5.5) and the final concentration in cell culture is 100 μM. d-Saccharic acid 1,4-lactone monohydrate was dissolved in 0.1 M sodium acetate buffer (pH 5.5), and the final concentration in cell culture is 100 μM. Before incubation, all cell media were adjusted to pH 5.5 by using 1 M HCl in order to mimic the microenvironment of inflammation. After incubation, the medium and cells were stored at −20 °C until further analysis. Experiments were performed at least with three replicates in three independent cultures.

### Measurement of β-glucuronidase activity

β-Glucuronidase (Helix pomatia-type H5, ≥400,000 Units/g solid) activity was determined by fluometrically monitoring the hydrolysis of 4-methylumbelliferyl-β-d-glucuronide (4MUgIA) according to the method described by (Bartholome et al. 2010) with some modifications. Briefly, the reaction mixture in a total volume of 140 μl contained 0.1 M sodium acetate buffer (pH 5.5), 2 mM 4MUgIA, and sample containing β-glucuronidase. The reaction was initiated by mixing 4MUgIA and the sample. The hydrolysis of 4MUgIA was measured in a thermostated plate reader (Spectra max m2, MDS, CA) at 37 °C and 320-/460-nm excitation/emission wavelengths. A standard curve of β-glucuronidase [0.4–400 Units/ml (U/ml)] was generated to quantitate formation of fluorescence in the presence of 4MUgIA. d-Saccharic acid 1,4-lactone monohydrate, a β-glucuronidase inhibitor, was used to inhibit β-glucuronidase activity.

For testing the interaction between B[a]P and β-glucuronidase, we used similar condition as mentioned above. A total volume of 140 μl contained 0.1 M sodium acetate buffer (pH 5.5), different concentrations of 4MUgIA (e.g. 500, 250, 100, 50, 10 and 1 μM), and 4 U/ml β-glucuronidase with additional 1 μl of 200 μM B[a]P or 1 μl DMSO. The measurement of fluorescence was performed for 10 h at 37 °C.

### HPLC fluorescence analysis of B[a]P and B[a]P metabolites

B[a]P and its metabolites were extracted from 5 ml cell medium by mixing with 1 ml ethylacetate for 20 min and followed by centrifugation (10 min, 980*g*). The top layer was transferred to a new tube. This procedure was repeated twice. The top layers were evaporated under nitrogen, and the residue was redissolved in 0.5 ml methanol (Biosolve Chemicals, Valkenswaard, the Netherlands). Samples were subsequently analysed by HPLC-FD using a Gynkotek P580A HPLC system (Separations Analytical Instruments, Hendrik-Ido-Ambacht, the Netherlands) consisting of a Spark SP830 autosampler (Spark Holland, Emmen, The Netherlands) and a Perkin Elmer LS-30 programmable fluorescence detector (Perkin Elmer, Foster City, CA, USA) operated at excitation/emission wavelengths 257/>350 nm. The samples were injected onto a Hypersil 5-μm ODS HPLC column (250 mm × 3 mm) (Supelco 54933, Bellefonte, PA, USA) with a flow rate of 0.5 ml/min. Separation was performed using a mixture of two mobile phases: A (100 % methanol) and B (40 % methanol in water) in the following multi-step gradient conditions: 0–5 min, 30/70 (A/B, v/v); 5–30 min, gradient from 30/70 (A/B, v/v) to 90/10 (A/B, v/v); 30–35 min, 90/10 (A/B, v/v); 35–37 min, gradient from 90/10 (A/B, v/v) to 30/70 (A/B, v/v); and 37–40 min, 30/70 (A/B, v/v). For quantitation of the specific metabolites, a standard mix which contained 50 ng/ml B[a]P-9,10-diol 50 ng/ml B[a]P-7,8-diol and 50 ng/ml 3-OH-B[a]P (Midwest Research Institute, Kansas City, MO, USA) were injected, and the area of each metabolite peak in the chromatogram was determined.

### Quantitative real-time PCR

Gene expression levels were measured by quantitative real-time reverse transcriptase PCR (RT-qPCR) using a MyiQ Single Colour real-time PCR detection system (BioRad, Veenendaal, The Netherlands). Total RNA was isolated and purified by using the RNeasy^®^ Mini Kit (Qiagen Westburg, Leusden, The Netherlands) in combination with DNase treatment (Qiagen). cDNA was generated from 500 ng total RNA by using the iScript™ cDNA synthesis kit protocol (BioRad). Primers were purchased from Operon (Leiden, The Netherlands) for the following genes: *β*-*actin*, *CYP1A1*, *CYP1B1* and *UGT1A6* [see (Schults et al. 2014)]. The reaction contained SYBR© Green Supermix (Bio-Ras), 5 μl (40 times diluted) cDNA and 0.3 μM primers in a total volume of 25 μl. PCR was conducted under the following condition: denaturation at 95 °C for 3 min, followed by 40 cycles of 95 °C for 10 s and 55 °C for 45 s. All PCR reactions included a cDNA dilution curve to assess PCR efficiency, and all reactions were followed by a melt curve (55–95 °C). Data were analysed by using MyiQ Software system (BioRad), and the amount of target cDNA in each sample was determined by a fractional PCR threshold cycle number (Ct value) and compared to the corresponding Ct value for the housekeeping gene β-actin. The relative gene expression level for each gene was calculated by using the 2^−ΔΔCt^ method (Livak and Schmittgen 2001).

## ^32^P-Postlabelling of B[a]P–DNA adducts

DNA harvested from cells was isolated using a phenol–chloroform–isoamylalcohol extraction procedure as described by Schults et al. (2013). Briefly, after incubation, cells were resuspended in 450 μl lysis buffer [10 mM Tris, 10 mM TEMPO, 1 mM EDTA and 1 % (w/v) sodium dodecyl sulphate (SDS); pH 8] and incubated with proteinase K (10 μg/ml) at 37 °C overnight. The mixture was extracted with 1 volume Tris-saturated phenol, 1 volume Tris-saturated phenol–chloroform–isoamyl alcohol (25:24:1 by volume), and 1 volume chloroform–isoamyl alcohol (24:1, v/v). The DNA was precipitated with 1/30 volume 3 M NaAc pH 5.2 and 2 volumes of cold 100 % ethanol. Precipitated DNA was washed with 70 % ethanol and dried under nitrogen. The DNA was dissolved in 2 mM Tris (pH 8.0) with final concentration 0.5 μg/μl.

DNA digestion and ^32^P-postlabelling were performed as described by Van Schooten et al. (1997). In short, DNA samples (10 μg) were digested with micrococcal nuclease (Sigma) (0.25 U/μl) and spleen phosphodiesterase (Sigma) (2 μg/μl) for 4 h at 37 °C in a total volume of 9.5 μl. For DNA adduct enrichment, samples were treated with nuclease P1 (Sigma) (2.5 μg/μl) at 37 °C for 30 min. The nuclease P1 reaction was terminated by addition of 1 µl 1 M Tris (pH 9.6). DNA adducts were subsequently labelled with [ɣ-^32^P]ATP (50 μCi/sample; PerkinElmer, Indianapolis) using T4-polynucleotide kinase (10 U/μl) for 30 min at 37 °C. The ^32^P-labelled adducts were separated on PEI–cellulose sheets (Machery Nagel, Düren, Germany) by multi-directional thin-layer chromatography (TLC).

The TLC sheets were scanned using Phosphor-Imaging technology (Fujifilm FLA-3000) and DNA adducts levels were calculated from two B[a]PDE–DNA standards with known adducts levels (1 adduct/10^6^ and 1 adduct/10^7^ nucleotides). The major B[a]P–DNA adduct that was used for quantitation purposes in both HepG2 and A549 cells migrated to the same position as the major adduct of the BPDE-DNA adduct standard. In addition, the B[a]PDE-DNA adduct levels were corrected for the amount of DNA in the sample which was assessed by HPLC–UV analysis.

### Statistical analysis

Data were expressed as mean ± standard error of the mean (SEM). Statistical analysis was performed using Graphpad Prism 6. To examine differences between the different treatments at each time point, a two-way analysis of variance test (ANOVA) with Bonferroni post hoc multiple comparison was used. Differences were considered to be statistically significant if the *p* value was less than 0.05 (*p* < 0.05).

## Results

### Activity of β-glucuronidase in lung and liver tissues of mice

Intranasal exposure of the mice to LPS resulted in an approximately twofold induction of β-glucuronidase activity in lung tissue compared to control (Table 1). Similarly, when LPS treatment was combined with B[a]P treatment, a significant 1.5-fold higher activity of β-glucuronidase was observed compared with the B[a]P-treated group without LPS. In addition, bronchoalveolar lavage fluid (BAL fluid) was collected, and the β-glucuronidase activity in BAL fluid was lower than in the tissues, but LPS treatment did result in a significant increase in β-glucuronidase when compared to animals that were not treated with LPS (i.e. irrespective of B[a]P exposure). On the other hand, in liver tissue a 1.2-fold lower activity of β-glucuronidase was observed in LPS-treated animals when compared to control and B[a]P-treated mice, respectively. These changes in β-glucuronidase activity after LPS treatment were in the range of 2–30 U/ml β-glucuronidase, and therefore, 4 U/ml was used as biologically relevant dose in the subsequent cell culture experiments (see Table 1).

**Table 1:** Activity of β-glucuronidase in mouse liver, lung tissues and BAL fluids

	Control (U/ml)	LPS (U/ml)	B[a]P (U/ml)	B[a]P and LPS (U/ml)
Liver cytosol	27.1 ± 1.5	23.3 ± 0.4*	27.6 ± 0.9	22.5 ± 1.0**
Lung cytosol	7.6 ± 0.8	15.4 ± 1.3*	7.4 ± 0.8	11.1 ± 1.3**
BAL fluids	2 ± 0.2	2.2 ± 0.1^#^	2.0 ± 0.1	2.2 ± 0.1^#^

* Significantly different from control animals (*p* < 0.05)

** Significantly different from B[a]P-treated animals (*p* < 0.05)

^#^ *p* < 0.05 if both LPS-treated groups were combined when compared to non-LPS-treated animals

### Phenotypes after B[a]P exposure with or without β-glucuronidase

#### Expression of CYP1A1 and CYP1B1

**Fig. 2:**
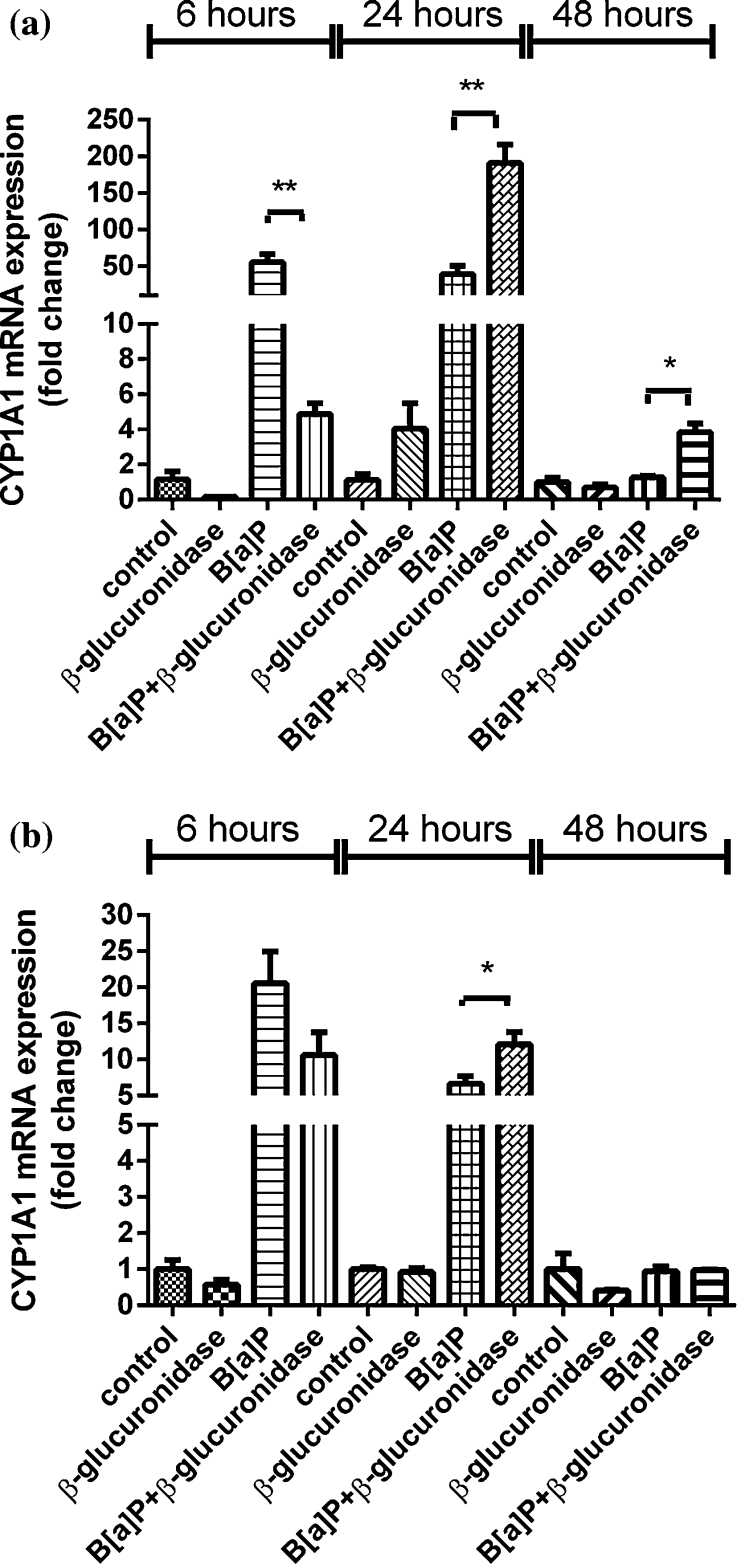
RT-qPCR analysis of gene expression *CYP1A1* in both A549 (**a**) and HepG2 (**b**) cells after exposure to β-glucuronidase and/or B[a]P. Cells were exposed to 1 μM B[a]P with or without 4 U/ml β-glucuronidase and harvested after the times indicated. Cells exposed to DMSO and sodium acetate buffer were used as a vehicle control. All values are given as the mean ± SEM (*n* = 4 per data point). (*p < 0.05; **p < 0.01; ***p < 0.001)

**Fig. 3:**
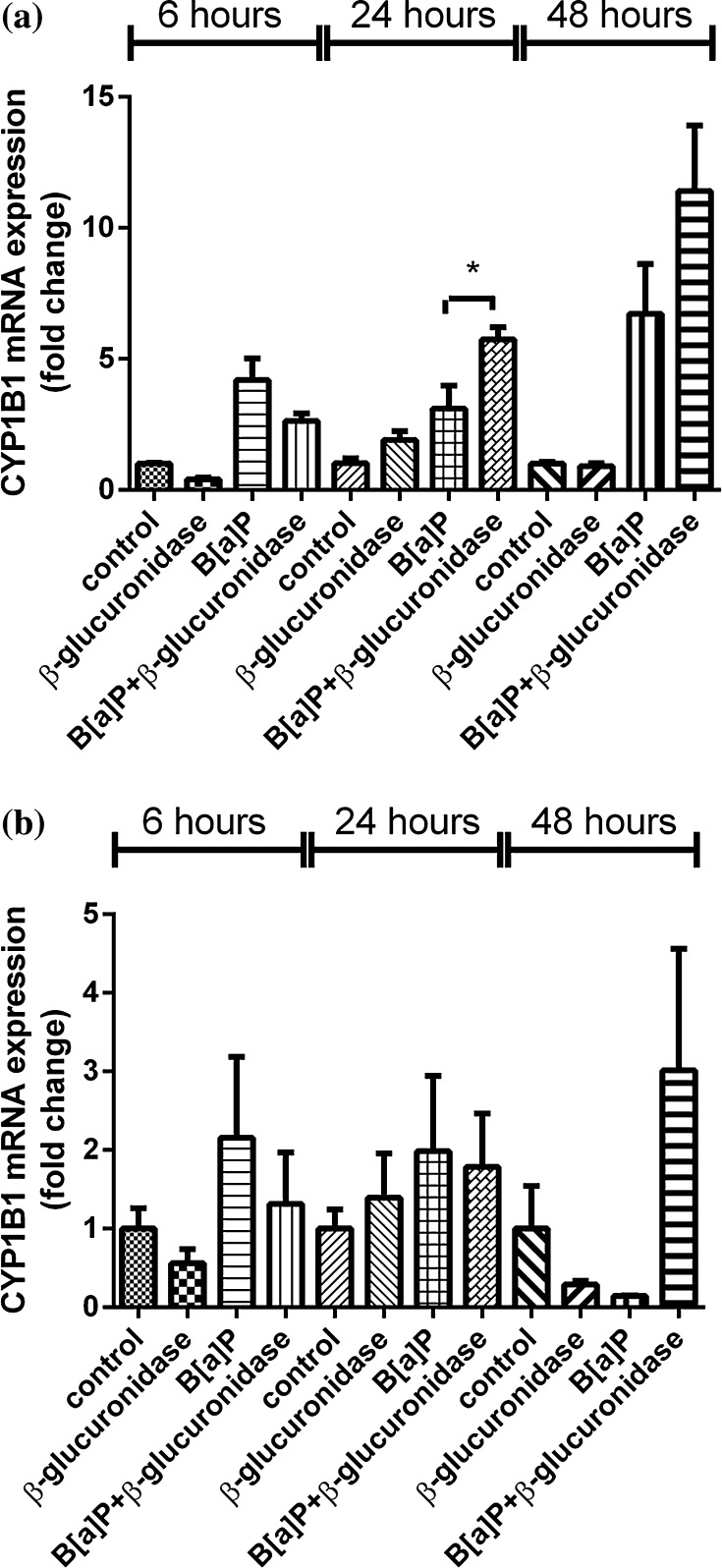
RT-qPCR analysis of gene expression *CYP1B* in both A549 (**a**) and HepG2 cells (**b**) after exposure to β-glucuronidase and/or B[a]P. Cells were exposed to 1 μM B[a]P with or without 4 U/ml β-glucuronidase and harvested after the times indicated. Cells exposed to DMSO and sodium acetate buffer were used as a vehicle control. All values are given as the mean ± SEM (*n* = 4 per data point). (*p < 0.05; **p < 0.01; ***p < 0.001)

Exposure to B[a]P significantly induced the expression of *CYP1A1* and *CYP1B1* (Figs. 2a, 3a). In A549 cells, the expression of these genes at 6 h after exposure was increased 56-fold and fivefold, respectively. Surprisingly, co-incubation with β-glucuronidase inhibited the induction of expression with approximately 50–90 %. Expression of *CYP1A1* and *CYP1B1* in cells that were treated with β-glucuronidase without additional exposure to B[a]P was also reduced when compared to the expression observed in control cells, but this difference did not reach statistical significance. At 24 h, the expression of *CYP1A1* and *CYP1B1* was induced by B[a]P to 39-fold and threefold, respectively. Surprisingly, the induction of both genes was now strongly increased by the presence of β-glucuronidase (191-fold and sixfold, respectively). At *t* = 48 h, the induction of *CYP1A1* and *CYP1B1* in cells that were co-exposed to β-glucuronidase remained higher than in cells that were only exposed to B[a]P. The same pattern of changes in gene expression of *CYP1A1* and *CYP1B1* by β-glucuronidase was observed in the absence of B[a]P, although less pronounced. Moreover, changes in gene expression of *CYP1A1* and *CYP1B1* were essentially similar in HepG2 cells (Figs. 2b, 3b), but the fold changes that were reached were lower than in A549 cells.

#### Expression of UGT1A6

**Fig. 4:**
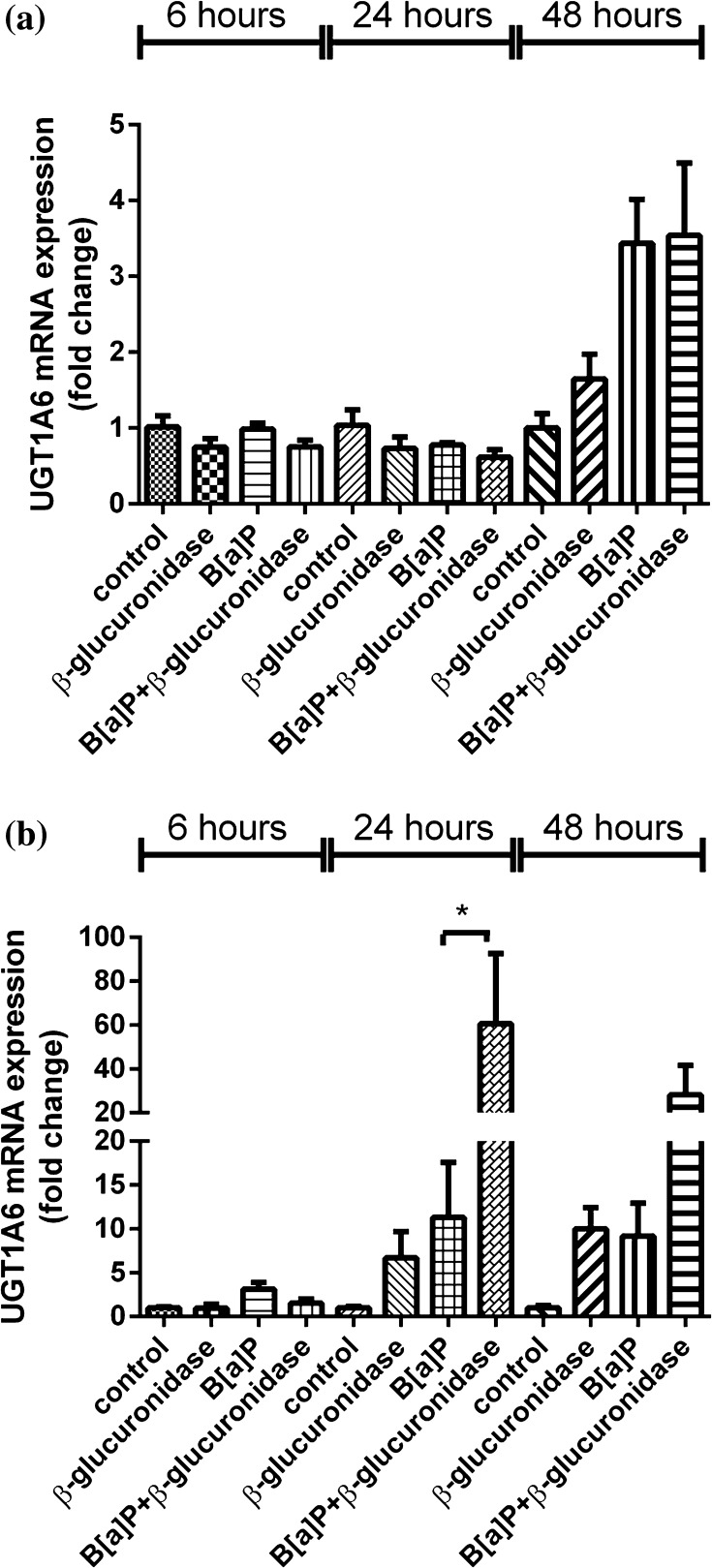
RT-qPCR analysis of gene expression *UGT1A6* in A549 (**a**) and HepG2 (**b**) cells after exposure to β-glucuronidase and/or B[a]P. Cells were exposed to 1 μM B[a]P with or without 4 U/ml β-glucuronidase and harvested after the times indicated. Cells exposed to DMSO and sodium acetate buffer were used as a vehicle control. All values are given as the mean ± SEM (*n* = 4 per data point). (*p < 0.05; **p < 0.01; ***p < 0.001)

As shown in Fig. 4b, in HepG2 cells the expression of UGT1A6 was threefold and 11-fold induced by B[a]P when compared to unexposed cells at 6 and 24 h after exposure, respectively. Addition of β-glucuronidase inhibited UGT1A6 expression after 6 h when compared to cells treated with B[a]P alone. However, a significant increase in UGT1A6 expression was observed 24 h after exposure (61-fold and sixfold compared to unexposed cells and only B[a]P-treated cells, respectively). At 48 h, the expression of UGT1A6 remained enhanced by B[a]P and B[a]P with additional β-glucuronidase, but the induction levels were three times lower (ninefold and 28-fold, respectively). In addition, a similar pattern of changes in gene expression of UGT1A6 were shown by β-glucuronidase in the absence of B[a]P.

On the other hand, in A549 cells, there was no significant induction or inhibition of UGT1A6 expression by B[a]P nor by β-glucuronidase, but at *t* = 48 h, all treatments induced the expression of UGT1A6 compared to unexposed cells (see Fig. 4a).

#### B[a]P and its metabolites

**Fig. 5:**
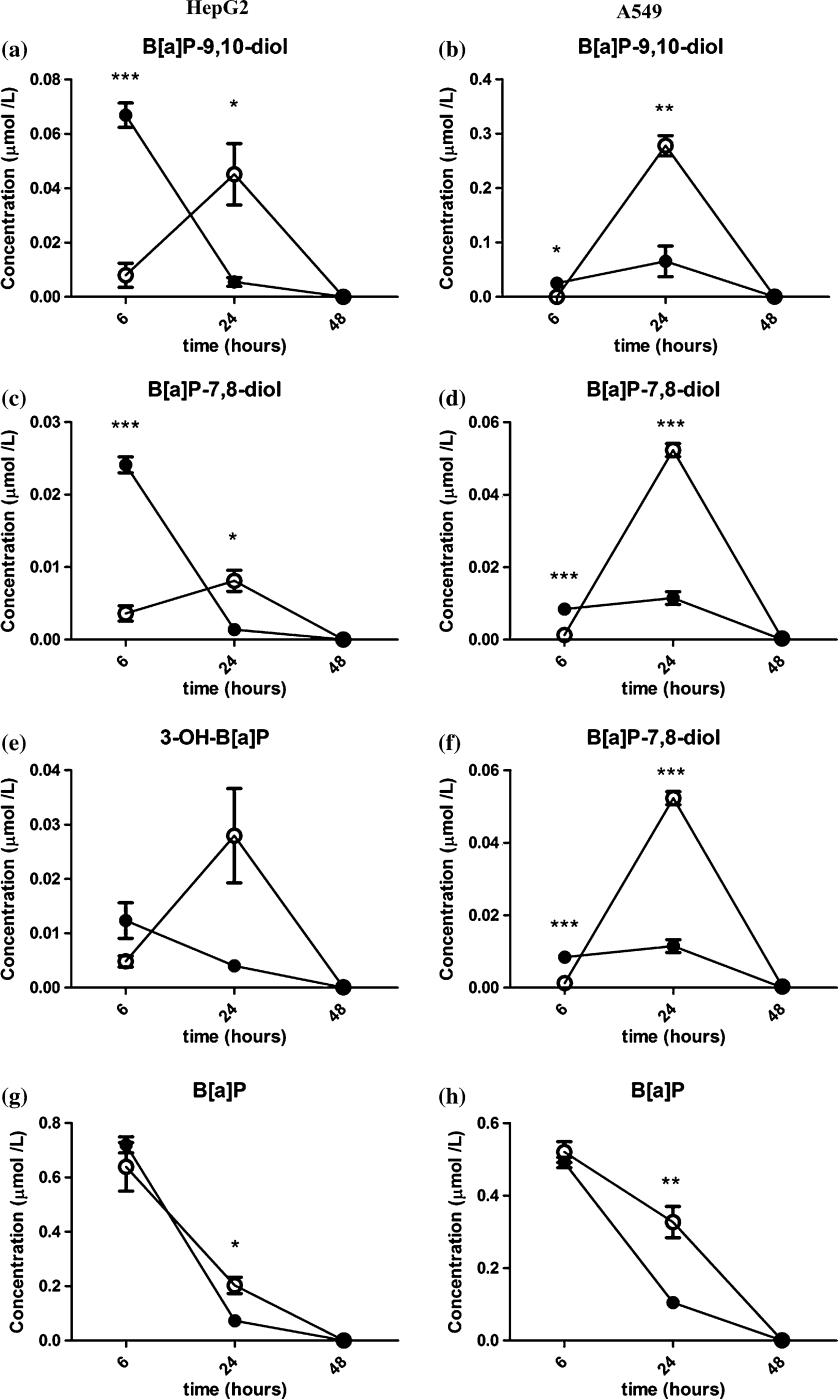
HPLC fluorescence analysis of B[a]P-7,8-diol, B[a]P-9,10-diol, 3-OH-B[a]P and B[a]P in HepG2 cells (left column) and A549 cells (*right column*) after exposure to β-glucuronidase and/or B[a]P. Cells were exposed to 1 μM B[a]P with or without 4 U/ml β-glucuronidase and cell medium was harvested after the time indicated. Cells exposed to DMSO and sodium acetate buffer were used as a vehicle control. All values are given as the mean ± SEM (*n* = 3 per data point). *Filled circle* without β-glucuronidase, *open circle* with β-glucuronidase

Extracellular B[a]P metabolite (B[a]P-7,8-diol and B[a]P-9,10-diol) concentrations of A549 cells that were exposed to 1 μM B[a]P with β-glucuronidase (10 µg/ml) for 6 h significantly decreased, when compared to A549 cells that were exposed to B[a]P only (Fig. 5, right column). However, when cells were exposed for 24 h, the concentrations of extracellular B[a]P metabolites in the medium of β-glucuronidase-treated cells significantly increased and were higher than in A549 cells that were exposed to B[a]P alone. At 48 h, the extracellular B[a]P metabolite levels were below the detection limit in both treatments (presence or absence of β-glucuronidase).

The results were essentially similar for HepG2 cells (Fig. 5, left column). However, the initial difference at *t* = 6 h was more pronounced, and statistically significant for B[a]P-7,8-diol and B[a]P-9,10-diol.

Moreover, the concentration of unmetabolized B[a]P in the medium showed similar time-dependent patterns in both cell lines (Fig. 5g, h). The concentration of unmetabolized B[a]P gradually declined with time. However, after 24 h of exposure, the concentration of the parent compound in A549 cells was about threefold higher (*p* < 0.05) in the samples that included β-glucuronidase than in cells without β-glucuronidase. For HepG2 cells, the concentration of B[a]P in the presence of β-glucuronidase was approximately twofold higher than in samples that were treated with B[a]P alone. For both cell lines, B[a]P was almost fully metabolized 48 h after exposure.

#### B[a]P–DNA adducts level

**Fig. 6:**
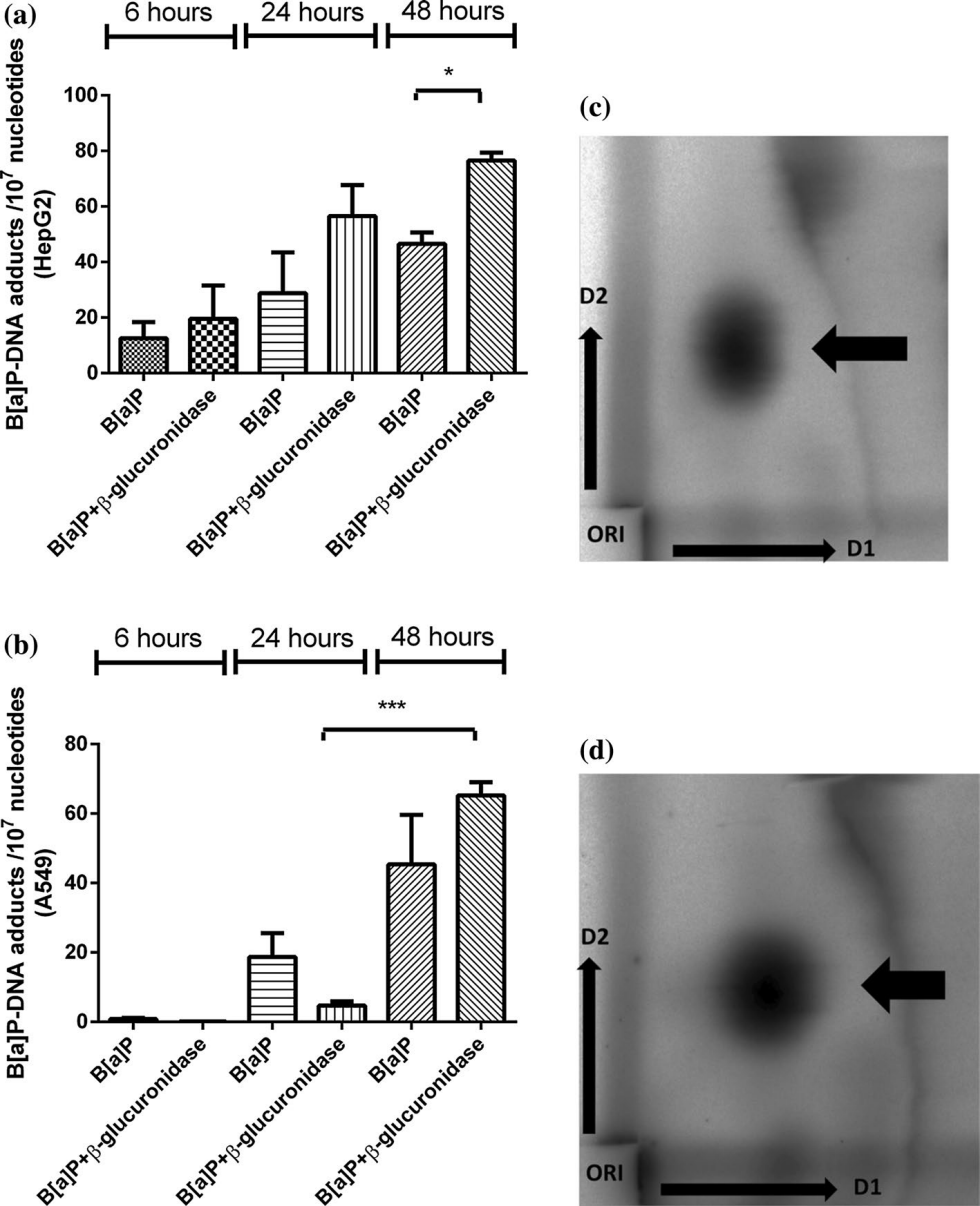
^32^P-Postlabelling analysis of DNA adducts level in HepG2 cells and A549 cells after exposure to B[a]P with or without β-glucuronidase. **a** HepG2 cells were exposed to 1 μM B[a]P with or without 10 μg/ml β-glucuronidase and harvest after the times indicated. **b** A549 cells were exposed to 1 μM B[a]P with or without 4 U/ml β-glucuronidase and harvest after the times indicated. Data are expressed as number of B[a]P–DNA adducts per 10^7^ nucleotides (*n* = 5 for HepG2 cells and *n* = 4 for A549 cells, mean ± SEM) (*p < 0.05; ***p < 0.001). Representative chromatograms obtained by ^32^P-postlabelling in HepG2 cells (**c**) and A549 cells (**d**). The adduct spot (*arrow*) that migrated during 2D-TLC to the same position as the major DNA adduct in a BPDE-DNA adduct standard was quantitated in all samples. Before phosphorimaging of the TLC plates the origin located at the bottom left-hand corner was excised

B[a]P exposure resulted in a time-dependent increase in B[a]P–DNA adduct levels in both cell lines (Fig. 6). However, the presence of β-glucuronidase altered the kinetics in which DNA adducts were formed: in A549 cells, at *t* = 6 and *t* = 24 h, DNA adduct levels were initially lower in cells that were treated with B[a]P and β-glucuronidase. However, a strong increase in DNA adduct levels from 5 adducts per 10^7^ nucleotides at *t* = 24 h to 65 adducts per 10^7^ nucleotides at 48 h (*p* < 0.0001), was found in β-glucuronidase-treated A549. This was not the case in the samples with B[a]P only. Consequently, at *t* = 48 h, B[a]P–DNA adduct levels were 1.4-fold higher in cells that were treated with β-glucuronidase compared to treatment with B[a]P only. In HepG2 cells, the presence of β-glucuronidase resulted in 1.5-fold, twofold, and 1.6-fold higher levels of B[a]P–DNA adducts at 6, 24, and 48 h, respectively, compared to the samples that were treated with B[a]P only (*p* < 0.05 at 48 h).

### Potential mechanisms

#### Can β-glucuronidase interact with B[a]P to prevent B[a]P from entering the cells?

**Fig. 7:**
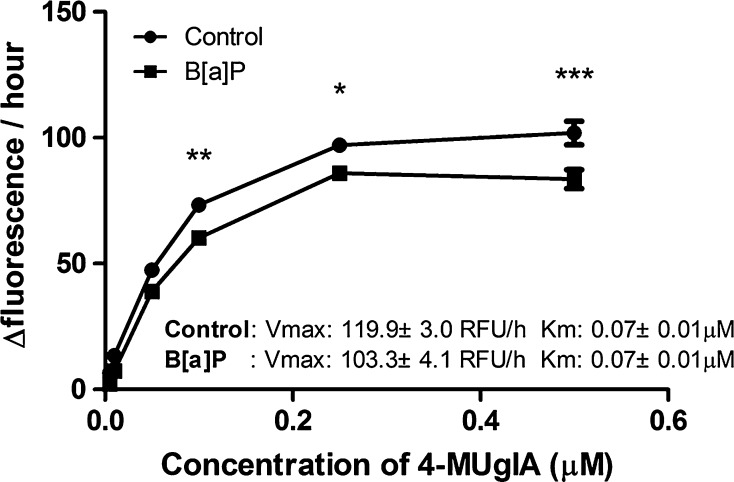
4MUgIA assay was applied to assess the possibility of B[a]P to bind to β-glucuronidase. A total volume of 140 μl contained 0.1 M sodium acetate buffer (pH 5.5), different concentration of 4MUgIA (e.g. 500, 250, 100, 50, 10 and 1 μM), and 4 U/ml β-glucuronidase with 1 μl of 200 μM B[a]P or 1 μl DMSO. The measurement of fluorescence [Relative Fluorescence Unit (RFU)] was performed for 10 h at 37 °C. B[a]P-treated samples were compared with control at each concentration, respectively. (*p < 0.05; **p < 0.01; ***p < 0.001)

Since B[a]P metabolism seems to be delayed, we studied whether B[a]P could temporarily bind to β-glucuronidase, which could prevent B[a]P from entering the cell. We assumed that binding of B[a]P to β-glucuronidase would interfere with β-glucuronidase activity. Therefore, we assessed the β-glucuronidase hydrolysis of 4MUgIA in the presence B[a]P (Fig. 7). At 37 °C for 10 h, 10 μg/ml of β-glucuronidase and 1 μM B[a]P were mixed with different concentration of substrate (4MUgIA). The addition of B[a]P lowered the changes in fluorescence units per hours compared with control. With increasing concentrations of 4MUgIA, the difference of the Δfluorescence/hour between these two groups became larger. This difference was significant at 100 μM (*p* < 0.01), 250 μM (*p* < 0.05) and 500 μM (*p* < 0.001) of 4MUgIA. The largest difference was observed at the highest concentration of 4MUgIA and was approximately 18 % lower than control. In addition, we performed Michaelis–Menten equation to determine the *V*_max_ and *K*_m_ for both reactions. Although the *K*_m_ in both reactions is same (0.07 ± 0.01 µM and 0.07 ± 0.01 µM for control and B[a]P-treated, respectively), the *V*_max_ in control is significantly larger than in the B[a]P-treated sample (*p* < 0.05). Therefore, we concluded that there is a non-competitive inhibition reaction.

#### Is the inhibitory effect of β-glucuronidase on CYP1A1 expression dependent on its activity?

**Fig. 8:**
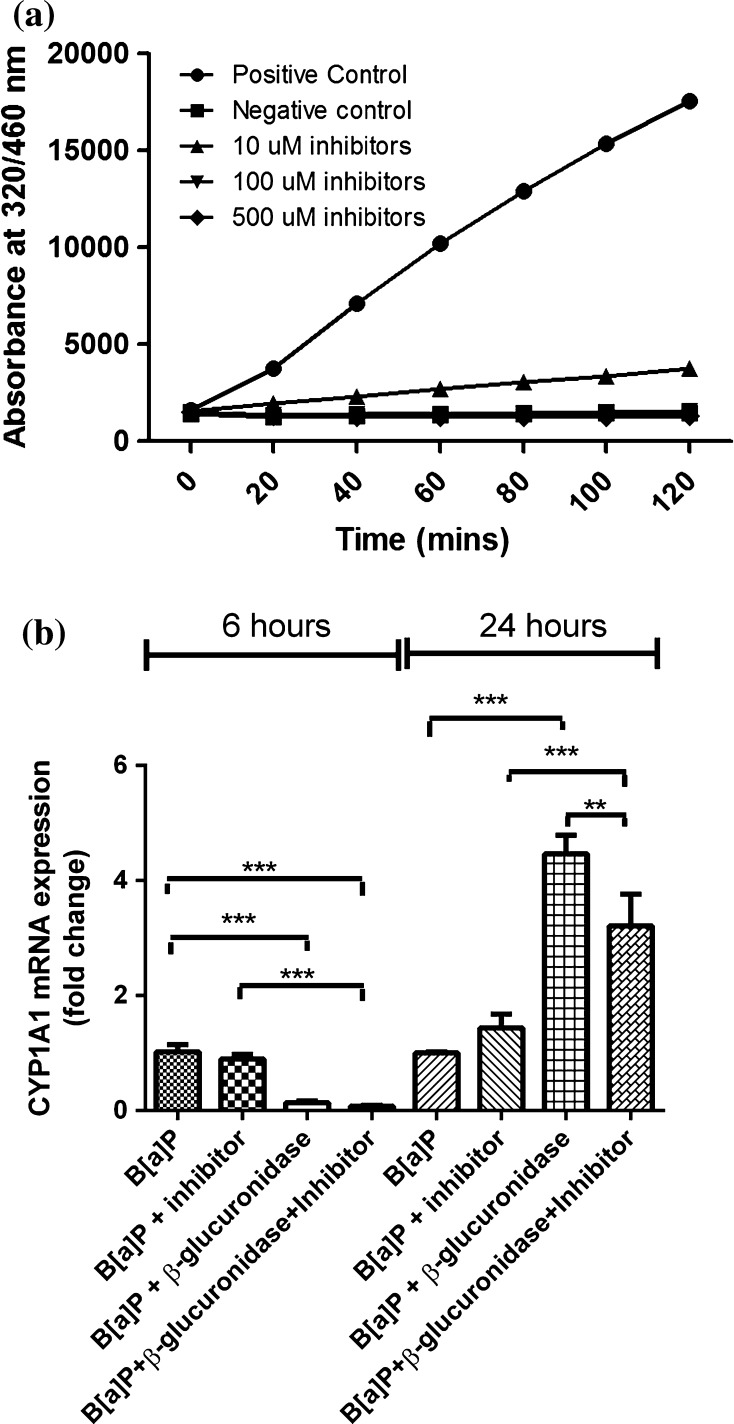
**a** 4MUgIA assay was applied to assess the inhibition of β-glucuronidase by d-saccharic acid 1,4-lactone monohydrate. A total volume of 140 μl contained 0.1 M sodium acetate buffer (pH 5.5), 2 mM 4MUgIA, 4 U/ml β-glucuronidase and different concentration of d-saccharic acid 1,4-lactone monohydrate (10 μM, 100 and 500 μM). 100 μM of d-saccharic acid 1,4-lactone monohydrate was used in the following incubation. **b** A549 cells were exposed to 1 μM B[a]P with or without β-glucuronidase, d-saccharic acid 1,4-lactone monohydrate for 6 and 24 h. Cells exposed to 1 μM B[a]P and sodium acetate buffer was used as a control. All values are given as the mean ± SEM (*n* = 4 per data point) (*p < 0.05; **p < 0.01; ***p < 0.001)

In order to gain further insight into the role of β-glucuronidase activity in influencing *CYP1A1* expression, an β-glucuronidase inhibitor (d-saccharic acid 1,4-lactone monohydrate) was added to the B[a]P and β-glucuronidase incubations in A549 cells (Fig. 8). A concentration of 100 μM fully inhibited β-glucuronidase activity (Fig. 8a). As shown in Fig. 8b, the presence of this inhibitor in incubations with B[a]P and β-glucuronidase did not change the inhibitory effect of β-glucuronidase on *CYP1A1* expression (0.13 ± 0.03 and 0.08 ± 0.01 in the absence or presence of inhibitor, respectively) at *t* = 6 h. On the contrary, at *t* = 24 h, the presence of this inhibitor significantly lowered *CYP1A1* expression when compared to cells that were exposed to B[a]P and β-glucuronidase without inhibitor (*p* < 0.01).

#### Involvement of the insulin-like growth factor 2/mannose-6-phosphate pathway

**Fig. 9:**
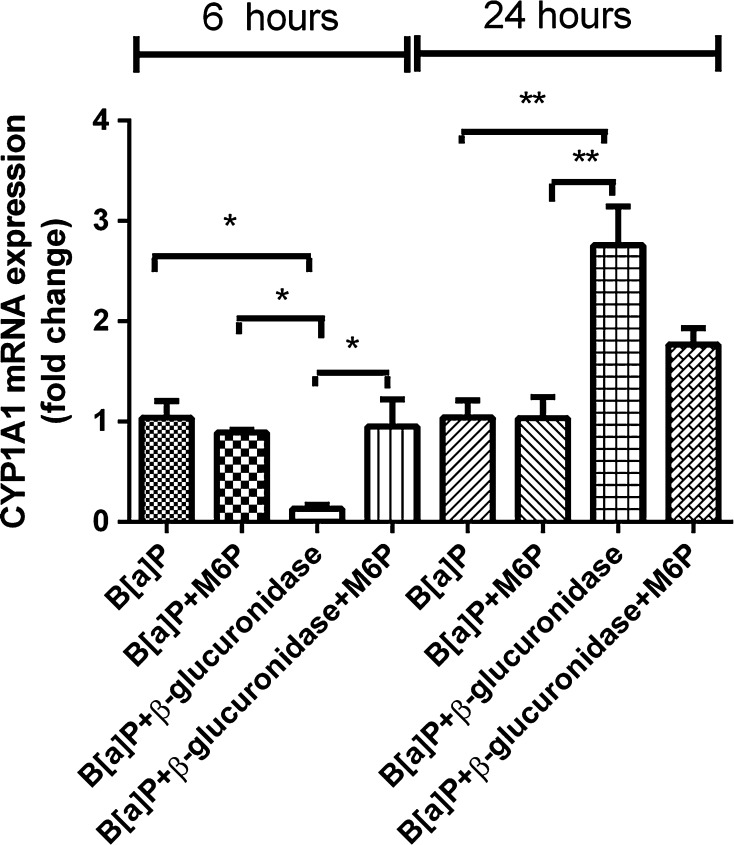
RT-qPCR analysis of gene expression *CYP1A1* in A549 cells after exposure to β-glucuronidase and B[a]P with the IGF2R inhibitor M6P. Cells were exposed to 1 μM B[a]P with or without 4 U/ml β-glucuronidase and with or without 100 μM M6P. Cells were harvested after the times indicated. Cells exposed to B[a]P and sodium acetate buffer were used as vehicle control. All values are given as the mean ± SEM (*n* = 4 per data point) (*p < 0.05; **p < 0.01; ***p < 0.001)

Extracellular enzymes like β-glucuronidase are known to bind to the mannose-6-phosphate (M6P) receptor (Gonzalez-Noriega et al. 2001), which is also known as IGF2 receptor. The IGF2 receptor can be inhibited by high concentrations of M6P (El-Shewy and Luttrell 2009). β-Glucuronidase inhibited the induction of *CYP1A1* expression by B[a]P at 6 h of incubation to approximately 10 % (Fig. 9), whereas in the presence of M6P, β-glucuronidase was unable to inhibit *CYP1A1* expression at 6 h. As shown previously, β-glucuronidase significantly induced *CYP1A1* expression at 24 h when co-incubated with B[a]P (*p* < 0.01). *CYP1A1* expression was still enhanced with additional M6P at 24 h, but the changes in expression were less pronounced.

## Discussion

It remains to be established how the cellular response to B[a]P is affected by the presence of extracellular β-glucuronidase, which is released during inflammation. In this study, we demonstrated that β-glucuronidase initially (6 h after exposure) inhibited gene expression of enzymes that are pivotal in B[a]P metabolism, including *CYP1A1*, *CYP1B1*, and *UGT1A6*. As a result, subsequent formation of B[a]P metabolites and the formation of B[a]P–DNA adducts was delayed in the presence of β-glucuronidase. However, at 24 h of exposure, *CYP* expression was significantly enhanced in β-glucuronidase-treated cells, probably because more B[a]P remained unmetabolized and β-glucuronidase converted B[a]P-derived glucuronide metabolites into active B[a]P metabolites. The higher concentration of active B[a]P metabolites continued to trigger the Ah receptor for gene expression of *CYP1A1* (in both cell lines) and *CYP1B1* (in A549 only). Consequently, the formation of active B[a]P metabolites and DNA adducts at *t* = 24 h was further increased in β-glucuronidase-treated cells. Because of this delayed metabolism of B[a]P, DNA adduct levels could accumulate in cells that were treated with β-glucuronidase and peaked at 48 h after the initial exposure.

A recent study observed that increased B[a]P–DNA adduct levels in mice that were exposed to B[a]P and intranasally instilled with LPS (Arlt et al. 2015). As LPS can induce an inflammatory response and stimulate the release of β-glucuronidase from neutrophils (Basinska and Florianczyk 2003; Ngkelo et al. 2012), we determined the β-glucuronidase activity in lung and liver tissues from these mice (Table 1). Indeed, β-glucuronidase activity was significantly enhanced in lung and BAL fluid of all LPS-treated animals when compared to the control group. On the other hand, we found that the β-glucuronidase activity was significantly decreased in liver tissue 3 days after being intranasally instilled with LPS. LPS treatment results in the recruitment of neutrophils from the liver to the lung, which could explain the lower β-glucuronidase activity in the liver after LPS treatment (Reutershan et al. 2005). The significant increase in β-glucuronidase activity in the lung samples by LPS treatment was approximately 4 U/ml, which we also subsequently used in our in vitro experiments.

B[a]P is known to bind to the AhR which stimulates its own metabolism by inducing the expression of *CYP1A1* and *CYP1B1* (Spink et al. 2002). *CYP1A1* and *CYP1B1* play an important role in both B[a]P activation and detoxification (Moserova et al. 2009), and B[a]P metabolites are further detoxified by glucuronidation. β-Glucuronidase will hydrolyse glucuronidated B[a]P metabolites and therefore will increase the concentration of active B[a]P metabolites (Shimoi and Nakayama 2005). Higher concentrations of these B[a]P metabolites (e.g. B[a]P-7,8-diol and B[a]P-9,10-diol) enhance the expression of *CYP1A1* and *CYP1B1* (Almahmeed et al. 2004; Spink et al. 2008). Therefore, we expected that addition of β-glucuronidase would increase *CYP1A1* and *CYP1B1* expression. However, we found that β-glucuronidase inhibited *CYP* expression shortly after B[a]P exposure (i.e. 6 h) in both A549 and HepG2 cells. This initial inhibition of gene expression by β-glucuronidase was independent of β-glucuronidase activity, because the β-glucuronidase inhibitor d-saccharic acid 1,4-lactone monohydrate did not change the results. Several studies report the binding of β-glucuronidase to insulin-like growth factor 2 receptors (IGF2R), which are located in the cell membrane (Gonzalez-Noriega and Michalak 2001; Urayama et al. 2004; Vogler et al. 2005). Moreover, IGF2 induced AhR in MCF-7 cells (Tomblin and Salisbury 2014). Therefore, we studied the involvement of IGF2R signalling by adding its inhibitor mannose-6-phosphate (M6P), and we found that the inhibitory effect of β-glucuronidase on CYP expression was also blocked. Therefore, we suggest a connection between IGF2R as receptor of extracellular β-glucuronidase and intracellular AhR-signalling after B[a]P exposure.

In addition, *UGT1A6* can be induced by AhR ligands in order to detoxify reactive B[a]P derivatives (Jin et al. 1993). UGT1A6 is an important enzyme for the detoxification of B[a]P metabolites and is predominantly located in human liver nuclear membranes (Radominska-Pandya et al. 2002; Zheng et al. 2002). This could explain the different kinetics of expression of *UGT1A6* between the two cell lines in this study. Hence, our mRNA expression data of *UGT1A6* exhibited a similar pattern as *CYP1A1* in HepG2 cells, but not in A549 cells. It is known that lung cells have a lower expression of UGT’s than liver cells (Ohno and Nakajin 2009), and therefore, it was expected that the effects of β-glucuronidase on the metabolism of B[a]P would be more pronounced in the liver-derived HepG2 cells than in the lung-derived A549 cells. Indeed, B[a]P–DNA adducts were already higher in β-glucuronidase-treated HepG2 cells at *t* = 24 h, whereas in A549 cells an additional 24 h was needed for further accumulation of DNA adducts. At the 24-h time point, the effects of β-glucuronidase on *CYP* expression were at least partly dependent on β-glucuronidase activity. Thus, we suggest that de-glucuronidated metabolites of B[a]P in combination with the higher concentrations of unmetabolized B[a]P, continued to trigger the Ah receptor and the subsequent expression of *CYP1A1* and *CYP1B1*. Therefore, our results indicate that there are two possible underlying mechanisms resulting in altered B[a]P metabolism and subsequent DNA adduct formation; (1) changes in gene expression by IGF2 signalling and (2) de-glucuronidation of glucuronidated metabolites.

Moreover, we studied whether B[a]P could temporarily bind to β-glucuronidase which would postpone B[a]P from entering the cell. Indeed, the presence of B[a]P decreased the capacity of β-glucuronidase to deconjugate 4MUgIA, suggesting an interaction between β-glucuronidase and B[a]P. However, this effect is unlikely to explain the present data, because the changes in β-glucuronidase activity by B[a]P are relatively small (<20 %), and in our experiments B[a]P is added in excess. However, this interaction may become more relevant at sites of inflammation and low B[a]P exposure.

Since CYP1A1 is considered to be the major enzyme for the activation and detoxification of B[a]P (Arlt et al. 2008), our measured B[a]P metabolites, including B[a]P-7,8-diol, paralleled the pattern of *CYP1A1* expression in both cell lines (HepG2 and A549). In addition, it is known that inhibition of *CYP1A1* decreased B[a]P–DNA adduct formation in vitro (Endo et al. 2008), but not in vivo (Ma and Lu 2007). However, we showed that with additional β-glucuronidase, the metabolism of B[a]P is delayed, which prolonged the effective exposure of cells to unmetabolized B[a]P, ultimately producing more toxic metabolites instead of excretable derivatives of B[a]P. A study using Cyp1a1(‒/‒) mice revealed that slower metabolic clearance of B[a]P may indeed lead to greater formation of B[a]P-mediated DNA adducts (Uno et al. 2001).

In summary, in this study we showed that β-glucuronidase alters the cellular response towards B[a]P by changing gene expression of *CYP1A1* in both lung- and liver-derived cells, ultimately causing higher DNA adduct levels. Moreover, we identified that β-glucuronidase may bind to IGF2R, thereby delaying B[a]P metabolism. This study exemplifies the complexity of the effect of inflammation on B[a]P-induced carcinogenesis, which deserves further attention.
